# The Principles and Practice of Obstetric Medicine and Surgery, in Reference to the Process of Parturition

**Published:** 1845-01

**Authors:** 


### Art. XX.

*The Principles and Practice of Obstetric Medicine and Surgery, in reference to the Process of Parturition; with* 110 *Illustrations on Steel and Wood.* By F. H. Ramsbotham, m.d., Fellow of the Royal College of Physicians, &c. &c.—*London*, 1844. 8vo, pp. 756.

This new edition of Dr. Ramsbotham's valuable work forms one of the most complete and thoroughly useful treatises on midwifery with which we are acquainted. It is not a mere reprint of the first edition, but contains an original essay occupying nearly a hundred pages on the diseases of the pregnant and puerperal states. Dr. Ramsbotham's remarks on these subjects, though necessarily brief, display the same talent for observation and the same practical tact as were shown in the first edition of the work; of which a rather full notice appeared in this Journal for Oct. 1841. The entire treatise has undergone a careful revision, and a few additions have been made to it; but the supplementary essay contains the only new matter of any importance. The chapters on Puerperal mania, Puerperal fever, Hidrosis and Scarlet fever, contain many extremely valuable observations. The chapter on Hidrosis is particularly interesting and we regret extremely to be obliged to omit, for want of room, a long extract from it which we had transcribed for the printer. We must, however, recommend Dr. Ramsbotham's excellent account of this rather rare disease, to the special notice of the reader.

The amount of mischief discovered after death in cases when this disease has run to a fatal termination is far less than is met with in other forms of puerperal disease in which symptoms of a much more alarming character have existed. Dr. Blundell professed himself to be wholly unable to give any account of the morbid appearances to which it gives rise, and though Dr. Ramsbotham has had the opportunity of making several post-mortem examinations, he has never found traces of disease anywhere except in the lining membrane or within the veins of the uterus. But as he truly observes, by no means every case of uterine phlebitis has been attended by symptoms of hidrosis. For our own part we should be disposed to look upon hidrosis not as a specific malady, but as one of the many varieties of morbid action consequent on a diseased state of the circulating fluid. Dr. Ramsbotham seems inclined to reject this hypothesis as applied to that large class of diseases included under the generic term of puerperal fever; and consequently treats of hysteritis, peritonitis, acute tympanitis, and of a fever allied to typhus, and of hidrosis, almost as if they were essentially different affections. He expresses himself as dissatisfied with the term puerperal fever, since he conceives that its use leads the young practititioner to confound together, both in description and in treatment, very dissimilar diseases. It does not, however, appear to us, that anything would be gained by discarding the term puerperal fever, which we regard as distinctive of a disease peculiar to lying-in women, attended by various local lesions, but always associated with febrile disturbance. Inflammation of the womb or the peritoneum in a lying-in woman does not of necessity imply the existence of puerperal fever, and the fever may run a fatal course and yet leave behind it no local lesion such as our modes of investigation are at present able to detect. No hypothesis seems to us so likely to unravel many of the abstruser questions of medical science, as one the foundation of which shall be laid in a well-understood humoral pathology. This opinion, too, seems further substantiated by the fact that the histories of most epidemics of puerperal fever, contain accounts of cases closely resembling hidrosis. Many such for instance occurred in the Dublin epidemic in the year 1810, and in 1836 at the lying-in hospital of Vienna. But we must not wander into these inquiries.

Dr. Ramsbotham's notice of the typhoid fever which sometimes occurs in puerperal women contains a striking illustration of the evils of treating a disease according to its name, and without proper attention to the peculiarities of its symptoms. He was summoned to see the wife of a medical friend who lived a short distance from town; she having been seized with typhus fever on the day after a perfectly natural labour. Her husband designated the disease puerperal fever, and acting upon preconceived notions was very desirous of bleeding her; but to this Dr. Ramsbotham was opposed, and stated it to be his opinion that the loss of a few ounces of blood would irrecoverably depress her. A small number of leeches were applied to the temples, and a purgative wasgiven, which was followed by the expulsion of a large quantity of fæces, and soon succeeded by great relief. The next day, however, an old practitioner, who was a relative of the patient's husband, was called in, and pronouncing the disease to be puerperal fever, expressed his surprise at Dr. Ramsbotham's remissness in not having bled her. He placed her in the half recumbent posture and bled her; but before six ounces of blood were drawn, she fainted, and died within four or five hours after the bleeding, never having rallied from the impression first produced. This fever was highly contagious, and having been communicated to three other patients, all of whom were bled by the medical practitioners who attended them, death in each instance occurred a few hours after the bleeding. The evil of the depletory treatment having been now fully recognized, no more patients were depleted, and no more deaths took place.

The extreme peril attendant on scarlatina in puerperal women is very forcibly dwelt on by the author, who states that out of a very considerable number of persons whom he has known to be attacked by it, only two recovered. There is indeed a peculiar malignancy about the disease when lying-in women become its subjects which is almost unaccountable. A striking instance of this, not mentioned by Dr. Ramsbotham, was afforded during the prevalence of scarlatina at Vienna in 1799 and 1800. The epidemic was characterized by its extreme mildness, except when it attacked puerperal women, who both in the lying-in hospital and out of it were carried off in large numbers on the fifth or sixth day. So fatal was it in the hospital, that the number of deaths rose from 9 to 49 in the course of the year. In the event of scarlatina occurring in the family of a person who is within one or two months of her confinement, Dr. Ramsbotham insists on her immediate removal from all communication with the infected parties, and on the continuance of this separation till at least a month after delivery. The author's experience has, we think, led him somewhat to overrate the fatality of scarlatina; there can, however, be no doubt that its attack exposes the pregnant or puerperal woman to great peril; and that even so strict a separation as he recommends cannot be regarded as a superfluous caution.

The chapters on abortion, and on the diseases of pregnancy, contain much very valuable information; but we have already given specimens of the work sufficient to justify our hearty recommendation of it as one of the best guides that the student or young practitioner can follow.

---

